# Ethics of antibiotic allergy

**DOI:** 10.1136/jme-2022-108648

**Published:** 2023-06-07

**Authors:** Yu Yi Xiang, George S Heriot, Euzebiusz Jamrozik

**Affiliations:** 1 Western Health, Footscray, Victoria, Australia; 2 Department of Infectious Diseases, The Peter Doherty Institute for Infection and Immunity, Melbourne, Victoria, Australia; 3 Ethox and Wellcome Centre for Ethics and Humanities, University of Oxford, Oxford, UK; 4 Royal Melbourne Hospital Department of Medicine, University of Melbourne, Melbourne, Victoria, Australia; 5 Monash Bioethics Centre, Monash University, Melbourne, Victoria, Australia

## Abstract

Antibiotic allergies are commonly reported among patients, but most do not experience reactions on rechallenge with the same agents. These reported allergies complicate management of infections in patients labelled as having penicillin allergy, including serious infections where penicillin-based antibiotics are the first-line (most effective and least toxic) treatment option. Allergy labels are rarely questioned in clinical practice, with many clinicians opting for inferior second-line antibiotics to avoid a perceived risk of allergy. Reported allergies thereby can have significant impacts on patients and public health, and present major ethical challenges. Antibiotic allergy testing has been described as a strategy to circumvent this dilemma, but it carries limitations that often make it less feasible in patients with acute infections or in community settings that lack access to allergy testing. This article provides an empirically informed ethical analysis of key considerations in this clinical dilemma, using *Staphylococcus aureus* bacteraemia in patients with penicillin allergies as a case study. We argue that prescribing first-line penicillin-based antibiotics to patients with reported allergies may often present a more favourable ratio of benefits to risks, and may therefore be more ethically appropriate than using second-line drugs. We recommend changes to policy-making, clinical research and medical education, in order to promote more ethically acceptable responses to antibiotic allergies than the status quo.

## Introduction

Many patients are labelled as being allergic to antibiotics, which leads to under-recognised ethical challenges. Consider this common clinical dilemma: a patient presents with a bacterial infection that warrants antibiotic treatment. The ‘first-line’ treatment for the infection is a penicillin antibiotic. This treatment is expected to be the best option for the patient (in terms of the balance of benefits, ie, chance of cure vs harms including antibiotic-related adverse effects) and public health (in terms of patient benefits vs public health harms, such as the development of resistant bacteria) compared to ‘second-line’ drugs. However, the patient’s medical record includes a label of penicillin allergy.

To treat this patient, the doctor has two main options:

1. Prescribe first-line (penicillin) treatment, despite the risk of allergic reaction, in order to offer the best chance of cure.
2. Prescribe second-line (non-penicillin) treatment, despite the lower chance of cure, in order to avoid an allergic reaction.

If the treatment is not urgent, doctors may also:

3. Perform allergy testing to determine whether the patient is truly allergic.

For now, assume that allergy testing is infeasible or the treatment is urgent. Deciding between first-line and second-line antibiotics can be a difficult ethical dilemma, because each option is associated with different benefits (eg, likelihood of cure) and risks (of allergic reaction and non-allergic harms), of varying magnitudes.

Physicians and patients almost always preference avoiding first-line therapy in the face of such dilemmas, due to a view that individuals with a label of antibiotic allergy face significant risks if exposed to the antibiotic (or class of antibiotics) in question.^1–3^ Physicians may also hold the view that causing harm (via a reaction to a prescribed drug) is less ethically acceptable than allowing harm to happen (by giving the patient treatment less likely to cure their infection).

Determining the ethically optimal treatment in such situations might, therefore, depend partly on how much moral weight doctors and patients attach to types of benefits and harms, which might be influenced by, inter alia, whether these outcomes are perceived to be caused by action or inaction. Yet ethical evaluations will also turn on empirical questions, since both the probability and the magnitude of the benefits and the harms involved will vary in different circumstances.

In this paper, we will argue that physicians and patients often give undue weight to risks related to antibiotic allergy. This results in second-line therapy being used too often, when (if antibiotic allergy risks were appropriately weighted) first-line therapy might offer a better overall balance of benefits and harms. To illustrate our argument with empirical data, we analyse the case study of bacteraemia caused by *Staphylococcus aureus* sensitive to methicillin (a type of penicillin antibiotic).

## The Epidemiology of Antibiotic Allergy

Approximately 10% of hospitalised patients in high-income countries report penicillin allergies.^1 4^ However, only around 5% of these have a ‘true’ allergy after allergy testing.^5^ Thus, less than 1% of people will have a confirmed allergic reaction to penicillin antibiotics,^4^ and approximately 0.001%–0.0005% will have anaphylaxis (ie, the most serious form of allergic reaction).^1 6^ This suggests that penicillin allergies are grossly over-reported, yet reports or labels of allergy strongly influence antibiotic prescription practices.^2^

Many false ‘allergies’ turn out to be non-immune-mediated reactions, as opposed to true, immune-mediated allergies.^7^ The severity of true allergies also wanes over time: 80% of allergic individuals become tolerant (ie, non-allergic) to penicillins after a decade.^8 9^ Moreover, many patients are inadvertently re-exposed to penicillin subsequent to acquiring an allergy label; if no reaction occurred on re-exposure there is likely no significant allergy.^7^

Using these data, scoring systems have been developed to enable healthcare workers to reclassify some patients as non-allergic based on a careful history of the reported ‘allergy’.^10^ Yet in practice even dubious allergy labels persist for long periods of time and are rarely reclassified.^11^

Patients with penicillin allergies therefore often receive second-line, rather than first-line antibiotics. Second-line drugs are typically associated with individual harms including lower cure rates^12 13^ and more non-allergy adverse drug reactions (ADRs),^1 12^ as well as public health harms including greater healthcare costs^14^ and increased prevalence of resistant bacteria.^15^

Penicillin allergies are one driver of healthcare-associated infections. Substituting first-line penicillins for second-line antibiotics can result in longer treatment durations, and greater disruption of the gut microbiome. Among other things, disruption of gut microflora increases the risk of *Clostridioides difficile* infection (CDI) risk (26% increase in incidence of CDI in a UK cohort, after adjusting for other CDI risk factors),^16^ which carries significant morbidity and mortality.^15 17^

## Case Study: *S. Aureus* Bacteraemia

*S. aureus* is a ubiquitous species of bacteria^18^ that causes skin and sometimes serious bloodstream infections (bacteraemia).^19^ *S. aureus* is often classified by its sensitivity to key antibiotics such as methicillin (a penicillin-based antibiotic); the two major types are methicillin-sensitive *S. aureus* (MSSA) and methicillin-resistant *S. aureus* (MRSA).^19^

MSSA bacteraemia is a common and serious clinical problem, causing deep-seated complications such as endocarditis (ie, heart valve infection).^12 19^ It also has mortality rates ranging from 15% to 50%, with frequent recurrences if treated inadequately.^19–21^ Up to 25% of MSSA bacteraemic patients report penicillin allergies.^4^ This is higher than the background prevalence of penicillin allergy, possibly reflecting a high average degree of prior healthcare and antibiotic exposure.

First-line treatment options for MSSA bacteraemia are primarily antistaphylococcal penicillins (such as nafcillin, oxacillin, and flucloxacillin) and cefazolin (also derived from penicillin), which all belong to the beta-lactam (ie, penicillin-derived) group of antibiotics.^i^ In patients with *reported* penicillin allergies, second-line antibiotics, such as vancomycin, are more likely to be used to avoid allergic reactions in those patients with *true* penicillin allergy.^3 4 12 22 23^ Vancomycin use results in around 20% lower MSSA bacteraemia cure rates, 2–3 times higher mortality rates compared with beta-lactams,^12 22^ and thus has serious consequences in terms of treatment failure.

Vancomycin use may also result in a higher risk of non-allergy adverse drug reactions (ADRs) than first-line options (table 1).^24^ Vancomycin also carries a risk of (non-penicillin) allergy that may be as high as first-line agents.^12^

**Figure 1:**
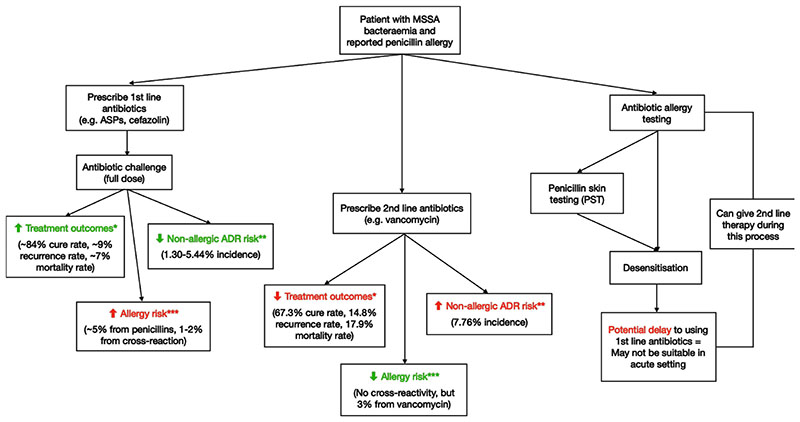
Flow chart with treatment options for *Staphylococcus aureus* bacteraemia with estimated risks and benefits. *Values retrieved from Blumenthal *et al*.^12^ **Values retrieved from Wynn *et al*^24^ and looks at total ADR incidence for each antibiotic. ***Values retrieved from Sacco *et al*.^5 12^ Note: Given the paucity of primary data on comparing different treatment approaches for MSSA bacteraemic patient cohorts with reported penicillin allergy, statistics for benefits and risk are only approximations adapted from various sources. ADR, adverse drug reaction; ASPs, antistaphylococcal penicillins; MSSA, methicillin-sensitive *Staphylococcus aureus*.

Figure 1 summarises the many potential benefits and risks physicians should arguably consider when making treatment decisions in MSSA bacteraemia patients labelled with penicillin allergy. In particular, the probability of causing harm through the use of penicillins is low, even with patients labelled with penicillin allergy.

## Ethical Analysis

The practice of avoiding the use of an antibiotic to which a patient has a reported allergy is widespread, even where this means opting for a second-line drug with inferior cure rates,^25 26^ and even for serious infections such as *S. aureus* bacteraemia.^12^ Below, we argue that such practices should be reformed: given a range of reasonable value weightings, it is often ethically preferable to treat patients with a first-line antibiotic even if they report an allergy to the drug in question. We offer several ways to deal with the ethical tensions and respond to potential objections.

While physicians’ ethical codes typically prioritise avoiding harming patients, exposing patients to risk cannot be absolutely proscribed, since a wide range of beneficial clinical activities (from surgery to drug prescription) involve risk to patients. Avoiding risk altogether would involve foregoing almost every benefit of modern medicine. In many cases, this would result in greater expected harms (in terms of lost benefits) to patients.

Suppose that prescribers make (formal or informal) assessments of expected benefit and harm at the time of antibiotic description. One approach might be to consider individual patient expected utility, where prescribers estimate the probability of different outcomes (based on published data and facts about a particular patient) and attach a utility weight to each outcome depending on how beneficial or harmful the prescriber thinks it may be for the patient. The expected utility is derived from adjusting utility weights according to their probability. While different outcomes may not be directly comparable (ie, they are incommensurable), and prescribers may have different estimates of the relevant benefits and harms, this at least provides a starting point for rational decisions and comparison of different clinical practices. We are interested in common situations where prescribers’ (and patients’) estimates of probabilities and outcomes diverge significantly from estimates derived from relevant population-level data.

Insofar as different harms and benefits are at least comparable, it may still be ethically appropriate for physicians to avoid acts that are more directly involved in causing harm than those that are more indirectly related to harm (ie, one possible interpretation of non-maleficence). One way this might occur is by attributing greater moral weight to particular harms (eg, drug allergies). By moral weight, we mean the increased importance given to a consideration over and above the probability and magnitude of the outcome (whether beneficial or harmful).^ii^ In the case of patients with antibiotic allergies, allergic reactions are highly salient to physicians and patients, and may, therefore, be given significant additional moral weight, which often results in altered treatment decisions. But is this reasonable?

The data presented above show that the label of an antibiotic allergy is a poor predictor of harm from allergic reactions on re-exposure to the antibiotic in question. As most patients labelled as allergic do not have a true allergy, the allergy label should be a relatively minor consideration in antibiotic prescription decision-making. Of course, it will sometimes be possible to identify patients who are at such high risk of severe allergic reaction that the antibiotic of interest should be avoided. For example, if the patient was exposed to penicillin 1 week ago and had anaphylaxis, it is far more likely that they are in the 5% of patients with true allergy and more likely that they will experience a severe reaction on re-exposure. There would, therefore, be stronger reasons to avoid prescribing the drug in question for that patient. But the more usual situation is that the patient’s previous reaction was many years ago and/or not serious or life-threatening. Given what we know about the low predictive value of such reports, almost all patients with an allergy label will not experience an allergic reaction on re-exposure.

In contrast, the use of second-line agents is associated with well-described harms. Some of these harms are caused by the drug, such as increased microbiome disruption and *C. difficile* infection, increased resistance (in the patient and, via transmission, in the community), and a risk of allergy as well as non-allergic ADRs due to second-line drugs.

Another set of harms relates to reduced cure rates (compared with first-line drugs). If there is a moral difference between harmful outcomes caused by prescribing a first-line drug (ie, an action perceived to directly cause allergic harm) and those caused by giving a second-line drug instead (thereby likely increasing the probability and/or magnitude of a severe infectious disease outcome, ‘indirectly’ harming the patient), then this might influence whether the harms of allergy outweigh those of reduced cure rates. However, if avoiding an allergy results in a clinician exposing patients to a much greater risk from treatment failure than the true allergy risk associated with a first-line drug, then one might think that this is an unreasonable practice.

In the case of S. aureus bacteraemia, the use of a second-line agent results in more than a doubling of mortality (from ~7% to 18%) from reduced cure rates. Moreover, the most common second-line agent (vancomycin) is itself associated with significant direct harms in terms of allergy and non-allergy ADRs.^24 27 28^

Given these data, one might think that most doctors’ clinical risk–benefit assessment should be overwhelmingly in favour of the use of first-line drugs in *S. aureus* bacteraemia, even in patients who report allergies to these drugs. This is because (1) the risk of allergy to first-line drugs is low even in those labelled with an allergy, (2) the risk of reduced cure with second-line drugs is high, and (3) the other direct risks associated with second-line drugs are similar to (and may sometimes be greater than) those associated with first-line drugs. One would have to give unreasonable weight to avoiding an allergic reaction in order to decide that consideration (1) outweighed considerations, (2) and (3). Moreover, insofar as one wishes to avoid direct harm to patients via prescribing decisions, to be consistent one must be concerned about (3) as well as (1). This makes it even less likely that (1) would outweigh (2) and (3), on a range of reasonable value weightings. Clinicians should, therefore, often use first-line antibiotics in patients with a reported allergy.

Why does this not occur in practice? There are several plausible reasons. First, physicians may be unaware of relevant data, and therefore, overestimate the risk of allergy, underestimate difference in effectiveness between first-line and second-line drugs, and/or underestimate the risks associated with second-line drugs.

Second, physicians may have a bias towards avoiding specific types of prescription-related harm, such as avoiding causing an allergic reaction. One reason may be that while severe allergic reactions are rare, the outcome can be serious or life-threatening. However, failing to cure a bacterial infection with second-line therapy can also be life-threatening, and patterns of practice suggest that prescribers often avoid an antibiotic even where the prior reported reaction is not consistent with true allergy. Physicians may also be influenced by professional norms and perhaps medicolegal concerns.^29^ Studies highlight that clinicians will occasionally use alternative treatments, even if inferior,^25^ in order to avoid (perceived) direct causal harm to patients.^30^

Third, physicians are subject to well-known cognitive biases that account for up to 75% of errors in medicine.^31^ Doctors may be more liable to make decisions based on cognitive biases in time-sensitive and critical scenarios, such as in a patient with bacteraemia.^32^ The most prominent cognitive bias in this situation is ‘base rate neglect’. The clinician may appreciate that a true penicillin allergy is rare, even in the context of a patient with an allergy label. However, the salience of allergy-related risks overrides this knowledge of the true base rate and the doctor may therefore opt for non-penicillin alternatives when treating such patients, regardless of the low probability of them having a true allergy. A related cognitive bias is ‘availability bias’: drug allergies may be more salient to the clinician because they have been made aware of severe allergies through medical education and/or recent treatment of patients with drug allergies.

Fourth, insofar as antibiotic prescribing decisions are discussed with patients, those patients who are aware of being labelled with an antibiotic allergy may strongly preference avoiding re-exposure to the antibiotic in question. These strong preferences may be rational in the minority of patients who have experienced recent, severe allergic reactions. But patients are also subject to cognitive biases, in many cases because they have been told in strong terms by doctors that, because they have an allergy, they must never again receive the antibiotic in question.^33^ Still, discussion with patients can sometimes result in consent to use first-line agents to which patients have a reported allergy.^33^ This is because patients might change their preferences once they are better informed about the poor predictive value of antibiotic allergy labels, the tendency of allergies to wane over time and/or the lower effectiveness and toxicity of second-line drugs.^iii^

### Objection: excessive responsibility for individual physicians

It might be objected that it is unreasonable to ask physicians to expose their patients to the (even small) risks of antibiotic allergy and to expose themselves to the (rare but significant) medicolegal consequences. It might also be objected that physicians often may not have the time for detailed discussion of relevant risks and benefits with patients (or patients’ next of kin) in time-critical situations in order to obtain consent for the use of first-line agents and the associated risk of antibiotic allergy.

We agree that not all of the responsibility for patterns of practice should rest with individual physicians. There is a clear need for changes in clinical guidelines and professional norms or models of practice to shift prescribing patterns in favour of more use of first-line agents, even in those labelled with an allergy. At a practical level, this might include, for example, multidisciplinary delabelling initiatives involving shared responsibility across healthcare teams.^34^ We would also recommend updating antibiotic advice from professional bodies (eg, medical colleges), changes in medical education, and further research (see below), all of which would help to alleviate the burden on individual physicians.

However, individual physicians arguably do have strong ethical reasons to reduce their own susceptibility to cognitive biases where this would result in improved patient outcomes.^31 32^ Moreover, there are also strong ethical reasons to make time to discuss risks and benefits with patients especially where patients’ preferences (and thus consent to a given treatment decision) would change if they were fully informed.

### Objection: allergy testing

One way of bypassing the dilemma raised at the start of this paper is to perform antibiotic allergy testing on all those who report an antibiotic allergy. This allows ‘delabelling’ (ie, removal of an antibiotic allergy label) for patients who test negative.^11^ For patients who test positive as allergic to the antibiotic in question, there is also the option of desensitisation, that is, the induction of tolerance to an antibiotic to which a patient is allergic by exposure to gradually increasing doses.^11 35^

Antibiotic allergy testing results in both individual and public health benefits. At the individual level, patients who are delabelled or desensitised benefit from (renewed) access to first-line antibiotics. Although patients who have true allergy do face some risks from allergy testing (these can be minimised by appropriate safeguards),^36–39^ the greater certainty regarding their allergic risk allows better management of future infections. At the level of public health, reducing the prevalence of antibiotic allergy labels results in less use of second-line drugs and thereby less antibiotic-related harms, resistance, and treatment costs.^12 40–43^

We support the expanded use of antibiotic allergy testing. However, such approaches face limitations including a scarcity of testing reagents^4^ and (at least at present) limited access to allergy testing due to facilities being concentrated in centres with subspecialist expertise and adequate resources.^4 11^

Access to allergy testing and desensitisation may also be limited in situations where a patient with an allergy label is already unwell with a serious infection such as *S. aureus* bacteraemia. However, if more patients in such situations are given first-line therapy this has the additional benefit of confirming or (in most cases) refuting the diagnosis of antibiotic allergy. Challenge with first-line agents during an infection may therefore obviate the need for future allergy testing. Still, some case reports suggest that desensitisation can be used successfully even during serious infections,^36–38^ and, this approach could potentially be used more widely, at least where it would be expected to result in better patient outcomes and/or be more cost-effective.^11^

## Policy Recommendations

Given the above analysis, we recommend changes to medical education, clinical practice, clinical guidelines and research. Physicians and other healthcare workers should be educated regarding the low veracity of antibiotic allergy labels and the harmful consequences of using second-line therapy and be encouraged to discuss these considerations with patients in the context of antibiotic prescribing decisions. Physicians should also be educated about cognitive biases and ethical reasoning, including reasons to avoid giving undue weight to avoiding a rare risk of serious allergy (eg, where this would involve foregoing much greater expected benefits and/or causing other harms).

Clinical practice guidelines should also change. Rather than the absolute avoidance of prescribing antibiotics to which a patient is thought to be allergic, guidelines should recommend that physicians weigh benefits and risks appropriately, including by being aware of the rarity of true serious allergy. As a result, practice should change: where appropriate, which is likely to be in the majority of cases, first-line antibiotics should be prescribed to most of the 10%–25% of patients who report an allergy to such drugs.^1 4^ Where available, referral for antibiotic allergy testing should be routine for all patients with allergy labels.

Finally, certain types of clinical research should be prioritised. Clinical trials of antibiotic options for common conditions should include patients with allergy where appropriate. There is also a need for rigorous published data on the allergic and non-allergic ADR risks associated with the use of all commonly prescribed antibiotics. Making physicians aware of the harms associated with first-line and second-line antibiotics (ideally framed in clinically useful terms such as the number needed to harm), and including these data in guidelines, will help physicians make ethically appropriate evidence-based risk-benefit assessments in clinical practice.

## Conclusions

It is often ethically appropriate to prescribe first-line antibiotics to patients who report an antibiotic allergy. We have illustrated the extent to which this may be expected to result in superior outcomes in the case of *S. aureus* bacteraemia and future work may aim to expand such analyses to other common clinically significant scenarios. Since antibiotic allergy labels are rarely veridical, there is likely broad scope for reform in clinical practice. While awaiting changes to clinical guidelines and medical education, doctors should make informed judgements about the risks and benefits in such scenarios, address their own cognitive biases and involve patients in decision-making. Antibiotic allergy testing should be much more widely used and empirical research should aim to clarify the benefits and risks of different antibiotic treatment options.

## Figures and Tables

**Table 1:** Non-allergic adverse drug reactions (ADRs) associated with antibiotics for *Staphylococcus aureus* infection

Antibiotic			ADR	Incidence (%)^24^	Estimated NNH*
Beta-lactams	ASPs	Nafcillin	Rash	2.17	18
			Diarrhoea	1.09	
			Nephrotoxicity	1.09	
			Nausea/vomiting	1.09	
			Total ADRs	5.44	
		Oxacillin	Nausea/vomiting	0.65	77
			Rash	0.65	
			Total ADRs	1.30	
	Cefazolin		Diarrhoea	0.99	40
			Rash	0.99	
			Leukopaenia	0.49	
			Total ADRs	2.47	
Vancomycin			Rash	3.88	13
			Leukopaenia	0.97	
			Nephrotoxicity	0.78	
			Diarrhoea	0.58	
			Nausea/vomiting	0.58	
			Others	0.97	
			Total ADRs	7.76	

* NNH (number need to harm) was estimated based on incidence of total ADRs, and in the absence of a control group in this retrospective cohort analysis. There appears to be a paucity of higher quality data, ie, randomised trials, reporting these antibiotic ADRs and calculations of NNH.

ASPs, antistaphylococcal penicillins; NNH, number needed to harm.

## Data Availability

All data relevant to the study are included in the article or uploaded as online supplemental information.

## Footnotes

i For the purposes of our analysis we focus on the choice between beta-lactams and vancomycin. We will not delve into the details of different beta-lactam agents (eg, different ASPs and cefazolin) although in practice they might have different advantages or disadvantages (see table 1). While the degree of cross-reactions in allergic responses to these agents may be low, many clinicians avoid all beta-lactams in patients with penicillin allergy (in line with the analysis in this paper).^22^

ii Of course, it may be difficult to separate individual empirical estimates of risk (including both the probability and magnitude of a harm) from moral evaluations. Some, but not all, divergence between individuals may be due to cognitive biases, discussed below, which may be at least partly remediable—while other differences in risk or benefit estimates may be more intractable. We thank an anonymous reviewer for pressing us on this point.

iii We thank an anonymous reviewer for pointing out that there may be a great deal to unpack regarding consent in such situations, especially at time of acute illness, which may for example fall short of idealistic notions of a perfectly informed patient, with full understanding, whose voluntary decision-making is not influenced by other people or factors. Although we give a minimal description here of how patients might change their views, further exploration of consent in such situations may be a rich area for future research.
